# The Role of Cardiac Rehabilitation in Promoting Lifestyle Modification Among Cardiovascular Patients: A Nationwide Cohort Study

**DOI:** 10.3390/healthcare12242553

**Published:** 2024-12-18

**Authors:** Chul Kim, Jung Hwa Hong, Jang Woo Lee

**Affiliations:** 1 Department of Rehabilitation Medicine, Inje University Sanggye Paik Hospital, Seoul 01757, Republic of Korea; josephck@naver.com; 2 Department of Research and Analysis, National Health Insurance Service Ilsan Hospital, Goyang 10444, Republic of Korea; jh_hong@nhimc.or.kr; 3 Department of Physical Medicine and Rehabilitation, National Health Insurance Service Ilsan Hospital, Goyang 10444, Republic of Korea

**Keywords:** cardiac rehabilitation, lifestyle, exercise, cohort study

## Abstract

Background: Cardiac rehabilitation (CR) is crucial for the secondary prevention of cardiovascular disease, primarily by promoting lifestyle modifications. However, its impact on lifestyle changes in the Korean population has not been well studied. This study analyzed data from the National Health Insurance Database to evaluate the effect of CR on lifestyle improvement in patients with cardiovascular disease. Methods: Patients who underwent coronary revascularization in 2017 were included. This study investigated whether the lifestyle habits of patients with cardiovascular disease who were physically inactive, obese, and smoked improved after coronary revascularization. To analyze whether CR affected each lifestyle factor, multiple regression analysis was performed, adjusting for socioeconomic and medical conditions as covariates. Results: CR had a significant effect on the acquisition of regular physical activity among physically inactive patients before revascularization (odds ratio [OR]: 1.205, 95% confidence interval [CI]: 1.046–1.389). However, CR did not have a significant impact on smoking cessation in smokers (OR: 1.172, 95% CI: 0.890–1.545) and weight reduction in patients with obesity (OR: 1.104, 95% CI: 0.977–1.248). Conclusion: This nationwide study showed that CR after coronary revascularization significantly improved physical activity in patients with cardiovascular disease. However, CR had no significant effect on smoking cessation or weight reduction, indicating a need for more comprehensive approaches to address these lifestyle factors.

## 1. Introduction

Cardiac rehabilitation (CR) is a well-designed management program based on secondary preventive effects such as exercise ability, quality of life, prevention of recurrence, and reduction of mortality in patients with cardiovascular disease (CVD) [1,2]. Accordingly, many clinical practice guidelines strongly recommend that patients with CVD participate in CR due to the high level of evidence supporting it [3].

As a secondary prevention program, comprehensive CR aims to improve physical, mental, nutritional, and vocational functioning [4]. As chronic diseases such as hypertension, diabetes, and hyperlipidemia and lifestyle habits such as smoking, obesity, and lack of exercise are risk factors for CVD, the purpose of CR is to correct habitual behaviors [5].

In Korea, CR has been reimbursed by the National Health Insurance Service since February 2017; this has led to increased interest in, and wider distribution of CR. CR insurance comprises three categories: Education, Evaluation, and Therapy. In CR Education, patients are educated to correct their lifestyles on a one-on-one basis in an independent space. CR Evaluation is a cardiovascular function evaluation package that includes the cardiopulmonary exercise test, which is implemented for cardiovascular risk stratification according to exercise, measurement of cardiorespiratory fitness and the establishment of a basis for an exercise prescription. CR Therapy involves supervised exercise training according to individualized exercise prescriptions based on the CR Evaluation results [6].

Secondary prevention through these CR programs is ultimately achieved through lifestyle modification [7,8]. However, in Korea, there is a scarcity of research on the practical effectiveness of CR programs in inducing lifestyle modifications. Given that CR has been reimbursed by the National Health Insurance Service since February 2017, using data from the National Health Insurance Database (NHID), we aimed to analyze whether lifestyle modifications in Korean patients were induced by CR.

## 2. Materials and Methods

### 2.1. Study Population

Using data from the NHID, we analyzed whether patients with CVD improved their lifestyles after receiving CR following coronary revascularization. Coronary revascularization included balloon angioplasty, thrombectomy, stent insertion, and bypass grafting; if one of the three prescriptions of CR was performed, the patient was considered to have undergone CR (Supplementary Table S1). Patients who underwent coronary artery revascularization between January and December 2017 were followed up until 2020. Lifestyle habits were analyzed using the National Health Examination data (included in the NHID) at the closest point before and after the time of revascularization. Patients who missed any health examination before or after revascularization, those who died within 1 month after revascularization, and those under 18 years of age were excluded from the analysis.

### 2.2. Outcomes

The correction of three lifestyle habits—physical activity, obesity, and smoking—was analyzed.

Physical activity was classified into three groups according to the International Physical Activity Questionnaire standard provided by the World Health Organization after converting exercise intensity into metabolic equivalent (MET) values in the National Health Examination Questionnaire [9]. High-intensity physical activity was calculated as 8.0 METs, medium-intensity physical activity was calculated as 4.0 METs, and low-intensity physical activity was calculated as 3.3 METs. The amount of physical activity per week was calculated by multiplying the number of weeks by the duration (min) of each physical activity. According to the calculated value, those with 599 METs·min or less were classified as the low-activity group, those with 600 METs·min or more and 2999 METs·min or less were classified as the moderate-activity group, and those with 3000 METs·min or more were classified as the high-activity group (Supplementary Figure S1) [10]. When a patient who was in the low-activity group changed to the moderate- or high-activity group after revascularization, it was defined as improved physical activity. Among the patients who were in the low-activity group before the onset of CVD, we analyzed whether CR affected the change in physical activity by comparing the cases of (1) remaining in the low-activity group even after the onset and (2) changing to the moderate- or high-intensity activity group.

Second, patients who were obese with a body mass index (BMI) of 25 kg/m^2^ or higher before the onset of CVD were compared with those who significantly reduced their weight after the onset and those who did not, and whether CR affected weight loss was analyzed. If patients with obesity lost more than 5% of their weight, significant weight loss was achieved [11].

Third, we compared whether patients who smoked before the onset of CVD continued smoking even after the onset and successfully quit smoking to analyze whether CR affected smoking cessation. Even a small amount of smoking increases the risk of CVD, indicating that smoking status is more important than the quantity of smoking [12]. Therefore, this study analyzed smoking cessation status rather than smoking quantity.

### 2.3. Identification of Other Influential Factors

Age was divided into five categories: (1) under the age of 40 years, (2) 40–49 years, (3) 50–59 years, (4) 60–69 years, and (5) >70 years. Residence was divided into urban and rural areas, and income level was divided into five categories based on the amount of health insurance premiums paid: (1) medical aid, (2) 25% or less, (3) 25–50%, (4) 50–75%, and (5) more than 75%. Hypertension, diabetes, and hyperlipidemia occurring before the onset of CVD were considered potential influencing factors. The Charlson Comorbidity Index (CCI) was used to correct the medical comorbidity (Supplementary Table S2) and was categorized as 0–2, 3–5, and 6 points or more (with higher score indicating a worse health condition).

The presence or absence of registered disabilities and musculoskeletal diseases was considered a factor that could affect participation in CR. Patients with physical disabilities, brain lesions, and mental disorders were grouped together, and patients without disabilities and those with disabilities other than physical disabilities, brain lesions, and mental disorders were grouped together. Physical disabilities, brain lesions, and mental disorders were classified into mild and severe disabilities, and patients were categorized into the following three categories: (1) individuals without disabilities and those with other disabilities, (2) those with mild disorders (physical disabilities, brain lesions, and mental disorders), and (3) those with severe disorders (physical disabilities, brain lesions, and mental disorders). Patients with musculoskeletal diseases were defined as those who took pain drugs (non-steroidal anti-inflammatory drugs, narcotic analgesics, gabapentin, and pregabalin) for at least 6 months in a year or who had undergone lower-limb arthroplasty or spine surgery.

After revascularization, rehospitalization within 28 days for any reason and non-hospitalization were distinguished. Moreover, if there was a coronary revascularization procedure or surgery within 10 years of coronary revascularization, it was defined as recurrence of CVD.

Alcohol consumption, smoking, and BMI data were extracted from the National Health Examination database at the closest point before the onset of CVD. Men were classified into a low-risk group (average less than 40 g/day, less than 60 g), medium-risk group (average more than 40 g/day), or high-risk group (average more than 60 g/day). Women were also classified into a low-risk group (average less than 20 g/day), medium-risk group (average more than 20 g/day, less than 40 g), or high-risk group (average more than 40 g/day) [13]. Smoking status was classified as current smokers, past smokers, and nonsmokers. BMI was classified into underweight (less than 18.5 kg/m^2^), normal weight (18.5–22.9 kg/m^2^), pre-obesity stage (23–24.9 kg/m^2^), and obesity (25 kg/m^2^ or more) according to Korean standards [14].

### 2.4. Statistical Analysis

The characteristics of patients with and without improvement in each lifestyle outcome were compared. The chi-square test was used to examine categorical variables for comparison of characteristics between the two groups. Multivariate logistic regression analysis was performed to analyze whether participation in CR affected lifestyle improvements. Although the confounders did not exhibit statistical differences in the univariable analysis, they were incorporated into the multivariable regression analysis to account for the diverse factors that could potentially impact the outcome. In each outcome analysis, the corresponding item was excluded from confounders, and only the remaining two variables were included. For example, when analyzing the effect of CR on changes in physical activity, only smoking and BMI were included as influencing factors, excluding the physical activity variable. SAS software (version 9.4; SAS Institute, Cary, NC, USA) was used for all analyses. A *p*-value of less than 0.05 was considered statistically significant.

### 2.5. Source of Data and Ethics Statement

This study used data sourced from the NHID (NHIS-2022-1-768) provided by the Korean National Health Insurance Service. This study was approved by the Institutional Review Board (IRB) of Sanggye Paik Hospital (IRB No. SGPAIK IRB 2020-04-004) on 16 April 2020. As all data were supplied by the Korean National Health Insurance Service and were fully anonymized, the IRB waived the requirement for informed consent.

## 3. Results

**Figure 1:**
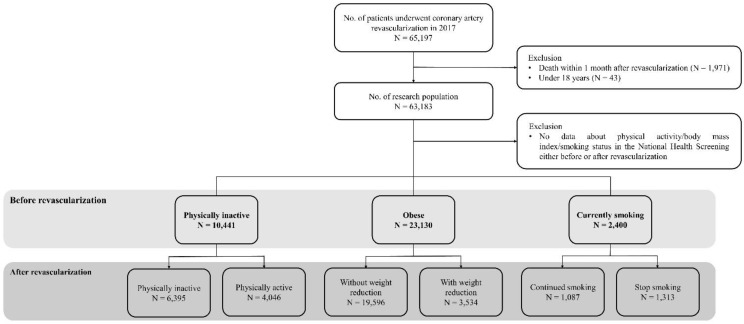
Flow chart of the study population.

In 2017, 65,197 patients underwent coronary revascularization. The total number of patients was 63,183, excluding those who died within 1 month after revascularization and those under 18 years of age. The physical activity, obesity, and smoking status of 10,441, 23,130, and 2400 patients were analyzed, respectively (Figure 1).

CR was associated with improved physical activity in patients with CVD who were physically inactive prior to revascularization. However, it had no significant effect on weight reduction in obese patients. Although univariable analysis indicated that CR had a significant role in smoking cessation, this effect was nullified in the multivariable analysis (Table 1).

### 3.1. Analysis of Patients Who Were Physically Inactive Prior to the Onset of CVD

Patients who were in the low-activity group (less than 600 METs·min) before the onset of CVD were compared (1) to patients in the low-activity group who remained in the same group after the onset of CVD and (2) to patients in the moderate-activity group (more than 600 METs·min and less than 3000 METs·min) or high-activity group (more than 3000 METs·min) after the onset of CVD.

Before the onset of CVD, 10,441 individuals were in the low-activity group, of which 6395 (61.2%) remained in the low-activity group even after the onset, while 4046 (38.8%) increased their physical activity after the onset of CVD, switching to the moderate- or high activity group. We found that 7.71% (493 patients) of those whose physical activity levels remained unchanged and 9.81% (397 patients) of those whose physical activity levels improved had participated in CR with a significant difference between the two groups. Except for hypertension, dyslipidemia, and BMI, all influencing factors showed a significant difference between the patient group remaining in the low-activity and the group that showed improvement (Supplementary Table S3).

We conducted multivariate logistic regression analysis to correct other influencing factors and found that CR, sex, income level, CCI, dyslipidemia, and musculoskeletal diseases influenced the change in physical activity level. The level of physical activity significantly improved in the patient group that underwent CR (*p* = 0.0105), and the level of physical activity was higher among men than among women. There was no improvement in the level of physical activity in the patient group with lower income compared to that in the high-income group, and there was no change in the level of physical activity in patients with high CCI values and musculoskeletal disease. However, the level of physical activity improved in patients with dyslipidemia (Supplementary Table S4).

### 3.2. Analysis of Patients Who Were Obese Prior to the Onset of CVD

Of the patients who were obese with a BMI of 25 kg/m^2^ or more before the onset of CVD, we compared (1) those with no significant weight loss and (2) those with a weight loss of 5% or more.

Of the 23,130 patients with obesity, 19,596 (84.7%) did not experience any significant weight loss after onset, while 3534 (15.3%) lost more than 5% of their weight after CVD. A total of 2271 (9.82%) of patients with obesity received CR. Of the patients who received CR, 9.77% (1915) of the patients did not have weight loss, and 10.07% (356) of the patients had weight loss of 5% or more, with no significant difference between the two groups. Except for CR, income level, and recurrence, all the indicators significantly differed between the group that did not lose weight and the group that succeeded in losing weight (Supplementary Table S5).

Multivariate logistic regression analysis was conducted to correct for other influencing factors and revealed that CR did not affect weight loss (*p* = 0.1015). However, a high CCI value, diabetes, musculoskeletal disease, and hospitalization within 28 days of discharge were associated with weight loss. In contrast, weight loss in patients with dyslipidemia, those who were current smokers, and those in the high physical activity group was minimal (Supplementary Table S6).

### 3.3. Analysis of Patients Who Were Smokers Prior to the Onset of CVD

Patients who were smokers before the onset of CVD were compared with those who (1) continued to smoke after the onset and (2) quit smoking.

There were 2400 smokers before the onset of CVD, of which 1087 (45.3%) continued to smoke after the onset, while more than half (1313; 54.7%) quit after the onset. Of the smokers, 253 (10.54%) received CR. Among them, 9.11% (99) patients who continued smoking and 11.73% (154) who succeeded in quitting smoking received CR with a significant difference in the rate of CR between the two groups (*p* = 0.0374). Other influencing factors that showed significant differences between the two groups included age, residence, income level, CCI, diabetes, dyslipidemia, musculoskeletal disease, alcohol consumption, physical activity, and recurrent CVD within 10 years (Supplementary Table S7).

According to the multivariate logistic regression analysis, CR did not affect smoking cessation (*p* = 0.2376). Smoking was more common in patients with the following characteristics: living in a rural area, low-income level, diabetes, dyslipidemia, alcohol consumption, and recurrent CVD within 10 years (Supplementary Table S8).

## 4. Discussion

Through an analysis of the Korean NHID, we examined whether CR had a statistically significant impact on lifestyle improvements in patients who underwent coronary revascularization due to CVD. The results showed that CR had a significant effect on improving physical activity in physically inactive patients. However, CR did not have a significant impact on weight reduction in obese patients and smoking cessation among smokers.

According to Chow et al. [15], the recurrence rate 6 months after acute myocardial infarction (AMI) decreases by 54% if patients faithfully adhere to exercise and diet programs and by 43% if they quit smoking. A study using big data from the Korean National Health Insurance Corporation also examined the 5-year prognosis of patients with AMI after discharge and found that the mortality rate and need for repeated revascularization were significantly reduced when regular exercise and smoking cessation were practiced after discharge [16]. Accumulating evidence highlights the critical role of lifestyle modifications, particularly physical activity, in improving long-term outcomes for patients with CVD.

The core goal of CR is to improve physical activity levels with aerobic exercise being the most important component [17]. Prescriptions for aerobic exercise should specify the frequency, intensity, time, and type. The level of exercise is determined by its frequency, intensity, and duration; increasing the level of exercise is the main goal of CR [18]. Although the history of CR in Korea is relatively short, the finding that CR had a positive effect on lifestyle, especially the physical activity of patients with CVD in this study, indicates that the CR system is functioning effectively.

Nevertheless, in this study, 61.2% of sedentary patients did not show improvement in their physical activity even after the onset of CVD, and 45.3% of smokers continued to smoke. Moreover, 84.7% of patients with obesity did not experience weight loss. Although 2017 marked the early stages of national health insurance coverage for CR, regrettably, only approximately 10% of patients participated in CR. However, the high possibility of lifestyle improvement in patients who have received CR highlights the need for more comprehensive CR programs and increased access for patients with CVD.

Unlike the effect of CR on the level of physical activity, CR did not have a significant effect on smoking cessation in smokers or weight reduction in patients with obesity. It is well known that smoking causes various health problems. More than half of the smokers succeeded in quitting smoking after the onset of CVD, indicating that patient awareness following disease onset may have had a stronger influence than the educational aspect of CR. Furthermore, considering that the offsetting effect of atherosclerotic disease due to smoking cessation does not appear immediately [19], continued intensive smoking cessation management is essential.

Similarly, it is well understood that obesity is closely related to CVD, and weight is determined by the balance between energy intake and expenditure [20]. Therefore, a healthy diet and exercise should be combined for effective weight loss [21]. In general, diet modification is more important than exercise for weight loss [22,23]. Previous studies have shown that exercise alone is not effective for weight reduction [24,25]. In this study, CR did not affect weight loss, whereas patients with unhealthy conditions, such as mild registered disabilities, diabetes, a high CCI score, musculoskeletal disease, and readmission within 28 days, experienced weight loss more often. Moreover, in many cases, weight loss was not achieved in patients in the highly active group. If the weight loss was significantly reduced after the onset of CVD, it may have been due to a pathological condition rather than intentional weight loss efforts. However, this distinction could not be made based on NHID data alone.

CR Evaluation and Therapy relate to actual exercise prescriptions, whereas CR Education encompasses physical activity and other CVD risk factors. In particular, smoking cessation and diet management are likely to have been intensively performed through CR Education rather than through CR Therapy. Therefore, the influence of each lifestyle modification may have differed depending on the type of CR the patient received. However, owing to the small number of patients who underwent CR, an analysis of each CR prescription code could not be performed.

This study has some limitations. First, the period of analysis after the initiation of national health insurance benefits for CR was short; therefore, it was not possible to analyze whether the change in physical activity after CR also affected the long-term prognosis. Second, the follow-up period after disease onset was short; therefore, it was not possible to analyze whether changes in lifestyle habits were maintained. Third, owing to the small number of patients who received CR, it was not possible to analyze by the component and intensity of CR. Finally, there was no information on changes in eating habits in the NHID; therefore, this could not be confirmed. When more data on CR prescriptions are available in the future, follow-up studies are needed from various perspectives, including the long-term prognosis.

## 5. Conclusions

This Korean national cohort study found that CR significantly improved physical activity levels in patients with CVD but had limited impact on smoking cessation and weight loss. These results highlight the importance of expanding CR programs to address a wider range of lifestyle modifications beyond physical activity, particularly in supporting smoking cessation and weight management, to maximize benefits for CVD patients.

## Figures and Tables

**Table 1:** The impact of cardiac rehabilitation on lifestyle improvement in patients with cardiovascular disease.

		Without Cardiac Rehabilitation, N (%)	With Cardiac Rehabilitation, N (%)	*p* for Univariable Analysis	Multivariable Analysis ^†^	
					OR (95% CI)	*p*
Physically inactive (N = 10,441)	No change in physical activity (N = 6395)	5902 (92.29)	493 (7.71)	0.0002 *	1.203 (1.044–1.386)	0.0105 *
	Improvement in physical activity (N = 4046)	3649 (90.19)	397 (9.81)			
Obese (N = 23,130)	Without weight reduction (N = 19,596)	17,681 (90.23)	1915 (9.77)	0.5797	1.108 (0.980–1.252)	0.1015
	With weight reduction (N = 3534)	3178 (89.93)	356 (10.07)			
Currently smoking (N = 2400)	Continued smoking (N = 1087)	988 (90.89)	99 (9.11)	0.0374 *	1.181 (0.896–1.556)	0.2376
	Stop smoking (N = 1313)	1159 (88.27)	154 (11.73)			

^†^ Adjusted by demographics, socioeconomic status, medical comorbidities, and lifestyles; * *p* < 0.05.

## Data Availability

The authors do not have the authority to distribute the original data, as it is derived from public data provided by the Korean National Health Insurance Service.

## Supplementary Materials

The following supporting information can be downloaded at https://www.mdpi.com/article/10.3390/healthcare12242553/s1, Figure S1: Physical activity grouping based on weekly exercise volume; Table S1. Claim codes of medical practices. Table S2. ICD-10 coding algorithms for Charlson Comorbidity Index. Table S3. Comparison between no change and improvement in physical activities after coronary revascularization in patients who were physically inactive before disease. Table S4. Multivariable logistic regression models for improvement of physical activity. Table S5. Comparison between obese patients who lost their weight and did not after coronary revascularization. Table S6. Multivariable logistic regression models for reduction in body weight among obese patients. Table S7. Comparison between smokers who continued and stopped smoking after coronary revascularization. Table S8. Multivariable logistic regression models for cessation of cigarette smoking.
