# Supplementary material for: The Role of Cardiac Rehabilitation in Promoting Lifestyle Modification Among Cardiovascular Patients: A Nationwide Cohort Study
Source: Healthcare (Basel). 2024 Dec 18;12(24):2553. doi: 10.3390/healthcare12242553 (PMC11675419; doi:10.3390/healthcare12242553)
Supplement: Supplementary file 1 [file healthcare-12-02553-s001.zip › healthcare-3287925-supplementary.pdf]

**Figure S1.** Physical activity grouping based on weekly exercise volume.

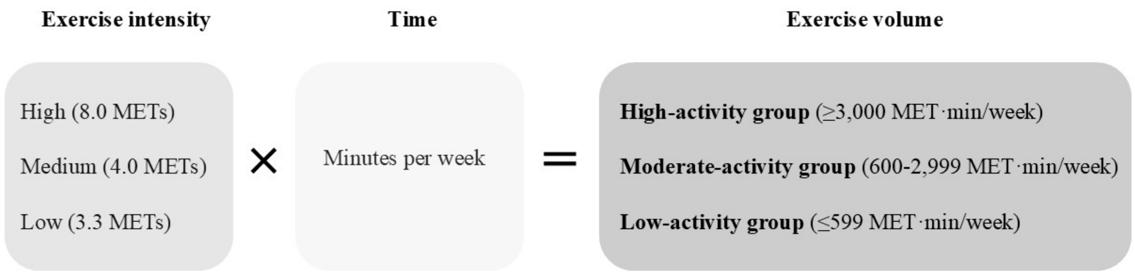


MET; metabolic equivalent

**Table S1.** Claim codes of medical practices.

| Medical practices | Claim codes |
|---|---|
| **Coronary revascularization** | |
| Balloon angioplasty | M6551x, M6552x, M6553x, M6554x |
| Thrombectomy | M6571x, M6572x |
| Stent insertion | M6561x, M6562x, M6563x, M6564x, M6565x, M6566x, M6567x |
| Bypass graft | O1640x, O1641x, O1642x, O1647x, O1648x, O1649x, OA640x, OA641x, OA647x, OA648x, OA649x |
| **Cardiac rehabilitation** | |
| Education | MM451 |
| Therapy | MM452 |
| Evaluation | MM453 |

**Table S2.** ICD-10 coding algorithms for Charlson Comorbidity Index.

| Comorbid conditions | ICD-10 codes | Assigned weighting |
|---|---|---|
| Acute myocardial infarction | I21.x, I22.x, I25.2 | 1 |
| Congestive heart failure | I09.9, I11.0, I13.0, I13.2, I25.2, I42.0, I42.5-I42.9, I43.x, I50.x, P29.0 | 1 |
| Peripheral vascular disease | I70.x, I71.x, I73.1, I73.8, I73.9, I77.1, I79.0, I79.2, K55.1, K55.8, K55.9, Z95.8, Z95.9 | 1 |
| Cerebrovascular disease | G45.x, G46.x, H34.0, I60.x-I69.x | 1 |
| Dementia | F00.x-F03.x, F05.1, G30.x, G31.1 | 1 |
| Chronic lung disease | I27.8, I27.9, J40.x-J47.x, J60.x-J67.x, J68.4, J70.1, J70.3 | 1 |
| Rheumatic disease | M05.x, M06.x, M31.5, M32.x-M34.x, M35.1, M35.3, M36.0 | 1 |
| Peptic ulcer | K25.x-K28.x | 1 |
| Mild liver disease | B18.x, K70.0-K70.3, K70.9, K71.3-K71.5, K71.7, K73.x, K74.x, K76.0, K76.2-K76.4, K76.8, K76.9, Z94.4 | 1 |
| Mild to moderate diabetes | E10.0, E10.1, E10.6, E10.8, E10.9, E11.0, E11.1, E11.6, E11.8, E11.9, E12.0, E12.1, E12.6, E12.8, E12.9, E13.0, E13.1, E13.6, E13.8, E13.9, E14.0, E14.1, E14.6, E14.8, E14.9 | 1 |

| | | |
|---|---|---|
| Diabetes with chronic complications | E10.2-E10.5, E10.7, E11.2-E11.5, E11.7, E12.2-E12.5, E12.7, E13.2-E13.5, E13.7, E14.2-E14.5, E14.7 | 2 |
| Hemiplegia or paraplegia | G04.1, G11.4, G80.1, G80.2, G81.x, G82.x, G83.0-G83.4, G83.9 | 2 |
| Kidney disease | I12.0, I13.1, N03.2-N03.7, N05.2-N05.7, N18.x, N19.x, N25.0, Z49.0-Z49.2, Z94.0, Z99.2 | 2 |
| Malignant tumors | C00.x-C26.x, C30.x-C34.x, C37.x-C41.x, C43.x, C45.x-C58.x, C60.x-C76.x, C81.x-C85.x, C88.x, C90.x-C97.x | 2 |
| Moderate to serious liver disease | I85.0, I85.9, I86.4, I98.2, K70.4, K71.1, K72.1, K72.9, K76.5, K76.6, K76.7 | 3 |
| Solid, metastatic tumor | C77.x-C80.x | 6 |
| AIDS | B20.x-B22.x, B24.x | 6 |

**Table S3.** Comparison between no change and improvement in physical activities after coronary revascularization in patients who were physically inactive before disease.

| | No change in physical activities (N=6,395), N (%) | Improvement in physical activities (N=4,046), N (%) | *P* |
|---|---|---|---|
| **Cardiac rehabilitation** | | | 0.0002 |
| No | 5,902 (92.29) | 3,649 (90.19) | |
| Yes | 493 (7.71) | 397 (9.81) | |
| **Sex** | | | <.0001 |
| Male | 4,406 (68.90) | 3,053 (75.48) | |
| Female | 1,989 (31.10) | 992 (24.52) | |
| Missing data | | 1 | |
| **Age (years)** | | | <.0001 |
| <40 | 53 (0.83) | 35 (0.87) | |
| ≥40 and <50 | 446 (6.97) | 344 (8.50) | |
| ≥50 and <60 | 1,358 (21.24) | 994 (24.57) | |
| >60 and <70 | 2,158 (33.75) | 1,464 (36.19) | |
| ≥70 | 2,380 (37.22) | 1,208 (29.86) | |
| Missing data | | 1 | |
| **Residence** | | | 0.0016 |
| Urban | 5,427 (85.22) | 3,529 (87.42) | |
| Rural | 941 (14.78) | 508 (12.58) | |
| Missing data | 27 | 9 | |
| **Income level** | | | 0.0019 |
| Medicaid | 220 (3.48) | 99 (2.48) | |
| 1st quantile (low) | 1,214 (19.20) | 720 (18.05) | |
| 2nd quantile | 1,138 (18.00) | 669 (16.77) | |
| 3rd quantile | 1,524 (24.11) | 989 (24.79) | |
| 4th quantile (high) | 2,226 (35.21) | 1,512 (37.90) | |
| Missing data | 73 | 57 | |
| **Registered disability** | | | 0.0027 |
| None or other disabilities | 5,697 (89.09) | 3,686 (91.10) | |
| Mild (brain/physical/mental) | 550 (8.60) | 292 (7.22) | |
| Severe (brain/physical/mental) | 148 (2.31) | 68 (1.68) | |
| **Charlson Comorbidity Index** | | | <.0001 |
| 0-2 | 1,817 (28.41) | 1,334 (32.97) | |
| 3-5 | 2,949 (46.11) | 1,861 (46.00) | |
| ≥6 | 1,629 (25.47) | 851 (21.03) | |

| | | | |
|---|---|---|---|
| **Hypertension** | | | 0.0928 |
| No | 1,063 (16.62) | 724 (17.89) | |
| Yes | 5,332 (83.38) | 3,322 (82.11) | |
| **Diabetes** | | | 0.0019 |
| No | 4,980 (77.87) | 3,254 (80.43) | |
| Yes | 1,415 (22.13) | 792 (19.57) | |
| **Dyslipidemia** | | | 0.0885 |
| No | 475 (7.43) | 265 (6.55) | |
| Yes | 5,920 (92.57) | 3,781 (93.45) | |
| **Musculoskeletal disorders** | | | <.0001 |
| No | 5,511 (86.18) | 3,664 (90.56) | |
| Yes | 884 (13.82) | 382 (9.44) | |
| **Smoking** | | | <.0001 |
| Never | 3,106 (48.57) | 1,770 (43.75) | |
| Ex-smoker | 1,416 (22.14) | 1,046 (25.85) | |
| Current smoker | 1,873 (29.29) | 1,230 (30.40) | |
| **Alcohol consumption** | | | <.0001 |
| None | 4,330 (67.71) | 2,578 (63.73) | |
| Low risk | 1,750 (27.37) | 1,275 (31.52) | |
| Moderate risk | 155 (2.42) | 103 (2.55) | |
| High risk | 160 (2.50) | 89 (2.20) | |
| Missing data | | 1 | |
| **Body mass index (kg/m$^2$)** | | | 0.0545 |
| <18.5 | 92 (1.44) | 41 (1.01) | |
| 18.5-22.9 | 1,640 (25.65) | 987 (24.39) | |
| 23-24.9 | 1,664 (26.02) | 1,120 (27.68) | |
| ≥25 | 2,998 (46.89) | 1,898 (46.91) | |
| Missing data | 1 | | |
| **Readmission before 28 days after discharge** | | | 0.0451 |
| No | 5,597 (87.52) | 3,594 (88.83) | |
| Yes | 798 (12.48) | 452 (11.17) | |
| **Recurrence within 10 years** | | | 0.0394 |
| No | 5,260 (82.25) | 3,391 (83.81) | |
| Yes | 1,135 (17.75) | 655 (16.19) | |

**Table S4.** Multivariable logistic regression models for improvement of physical activity.

| | OR | 95% CI | *P* |
|---|---|---|---|
| **Cardiac rehabilitation** | 1.203 | 1.044-1.386 | 0.0105 |
| **Sex** | | | |
| Male | 1.270 | 1.132-1.423 | <.0001 |
| Female | 1 (Ref) | | |
| **Age (years)** | | | |
| <40 | 1 (Ref) | | |
| ≥40 and <50 | 1.160 | 0.738-1.823 | 0.5202 |
| ≥50 and <60 | 1.176 | 0.758-1.824 | 0.4698 |
| >60 and <70 | 1.144 | 0.737-1.775 | 0.5494 |
| ≥70 | 0.895 | 0.574-1.396 | 0.6261 |
| **Residence** | | | |
| Urban | 1 (Ref) | | |
| Rural | 0.910 | 0.808-1.026 | 0.1231 |
| **Income level** | | | |

| | | | |
|---|---|---|---|
| Medicaid | 0.745 | 0.578-0.961 | 0.0232 |
| 1st quantile (low) | 0.875 | 0.779-0.982 | 0.0231 |
| 2nd quantile | 0.836 | 0.743-0.941 | 0.0030 |
| 3rd quantile | 0.932 | 0.839-1.036 | 0.1920 |
| 4th quantile (high) | 1 (Ref) | | |
| **Registered disability** | | | |
| None or other disabilities | 1 (Ref) | | |
| Mild (brain/physical/mental) | 0.896 | 0.770-1.042 | 0.1550 |
| Severe (brain/physical/mental) | 0.781 | 0.581-1.051 | 0.1028 |
| **Charlson Comorbidity Index** | | | |
| 0-2 | 1 (Ref) | | |
| 3-5 | 0.924 | 0.839-1.018 | 0.1084 |
| ≥6 | 0.822 | 0.715-0.945 | 0.0059 |
| **Hypertension** | 1.011 | 0.906-1.129 | 0.8846 |
| **Diabetes** | 1.024 | 0.907-1.155 | 0.7040 |
| **Dyslipidemia** | 1.214 | 1.033-1.427 | 0.0185 |
| **Musculoskeletal disorders** | 0.768 | 0.672-0.878 | <.0001 |
| **Smoking** | | | |
| Never | 1 (Ref) | | |
| Ex-smoker | 1.068 | 0.950-1.202 | 0.2720 |
| Current smoker | 0.894 | 0.796-1.055 | 0.0602 |
| **Alcohol consumption** | | | |
| None | 1 (Ref) | | |
| Low risk | 1.039 | 0.944-1.145 | 0.4322 |
| Moderate risk | 0.957 | 0.738-1.242 | 0.7428 |
| High risk | 0.823 | 0.627-1.079 | 0.1583 |
| **Body mass index (kg/m²)** | | | |
| <18.5 | 0.807 | 0.550-1.184 | 0.2724 |
| 18.5-22.9 | 1 (Ref) | | |
| 23-24.9 | 1.058 | 0.946-1.183 | 0.3243 |
| ≥25 | 0.994 | 0.898-1.100 | 0.9087 |
| **Readmission before 28 days after discharge** | 0.940 | 0.829-1.066 | 0.3372 |
| **Recurrence within 10 years** | 0.919 | 0.824-1.026 | 0.1324 |

**Table S5.** Comparison between obese patients who lost their weight and did not after coronary revascularization.

| . | **Without weight reduction (N=19,596), N (%)** | **With weight reduction (N=3,534), N (%)** | ***P*** |
|---|---|---|---|
| **Cardiac rehabilitation** | | | 0.5797 |
| No | 17,681 (90.23) | 3,178 (89.93) | |
| Yes | 1,915 (9.77) | 356 (10.07) | |
| **Sex** | | | <.0001 |
| Male | 15,397 (78.57) | 2,527 (71.53) | |
| Female | 4,199 (21.43) | 1,006 (28.47) | |
| Missing data | | 1 | |
| **Age (years)** | | | <.0001 |
| <40 | 208 (1.06) | 35 (0.99) | |
| ≥40 and <50 | 1,682 (8.58) | 271 (7.67) | |
| ≥50 and <60 | 4,953 (25.28) | 688 (19.47) | |
| >60 and <70 | 6,919 (35.31) | 1,156 (32.72) | |
| ≥70 | 5,834 (29.77) | 1,383 (39.15) | |

| | | | |
|---|---|---|---|
| Missing data | | 1 | |
| **Residence** | | | 0.0001 |
| Urban | 17,489 (89.45) | 3,072 (87.27) | |
| Rural | 2,062 (10.55) | 448 (12.73) | |
| Missing data | 45 | 14 | |
| **Income level** | | | 0.4242 |
| Medicaid | 464 (2.41) | 102 (2.93) | |
| 1st quantile (low) | 3,389 (17.57) | 612 (17.56) | |
| 2nd quantile | 3,352 (17.38) | 604 (17.33) | |
| 3rd quantile | 4,584 (23.76) | 804 (23.07) | |
| 4th quantile (high) | 7,503 (38.89) | 1,363 (39.11) | |
| Missing data | 304 | 49 | |
| **Registered disability** | | | <.0001 |
| None or other disabilities | 17,976 (91.73) | 3,159 (89.39) | |
| Mild (brain/physical/mental) | 1,305 (6.66) | 303 (8.57) | |
| Severe (brain/physical/mental) | 315 (1.61) | 72 (2.04) | |
| **Charlson Comorbidity Index** | | | <.0001 |
| 0-2 | 6,576 (33.56) | 929 (26.29) | |
| 3-5 | 8,982 (45.84) | 1,577 (44.62) | |
| ≥6 | 4,038 (20.61) | 1,028 (29.09) | |
| **Hypertension** | | | 0.0007 |
| No | 3,523 (17.98) | 552 (15.62) | |
| Yes | 16,073 (82.0) | 2,982 (84.38) | |
| **Diabetes** | | | <.0001 |
| No | 15,837 (80.82) | 2,610 (73.85) | |
| Yes | 3,759 (19.18) | 924 (26.15) | |
| **Dyslipidemia** | | | 0.0099 |
| No | 1,344 (6.86) | 285 (8.06) | |
| Yes | 18,252 (93.14) | 3,249 (91.94) | |
| **Musculoskeletal disorders** | | | <.0001 |
| No | 17,705 (90.35) | 3,028 (85.68) | |
| Yes | 1,891 (9.65) | 506 (14.32) | |
| **Smoking** | | | <.0001 |
| Never | 8,080 (41.24) | 1,750 (49.52) | |
| Ex-smoker | 5,794 (29.57) | 1,026 (29.03) | |
| Current smoker | 5,721 (29.20) | 758 (21.45) | |
| **Alcohol consumption** | | | <.0001 |
| None | 11,680 (59.60) | 2,278 (64.48) | |
| Low risk | 7,016 (35.80) | 1,112 (31.47) | |
| Moderate risk | 465 (2.37) | 66 (1.87) | |
| High risk | 435 (2.22) | 77 (2.18) | |
| Missing data | | 1 | |
| **Exercise volume (METs·min/week)** | | | 0.0016 |
| ≥3000 | 1,964 (10.02) | 307 (8.69) | |
| ≥600 and <3000 | 8,874 (45.28) | 1,544 (43.69) | |
| <600 | 8,758 (44.69) | 1,683 (47.62) | |
| **Readmission before 28 days after discharge** | | | <.0001 |
| No | 17,560 (89.61) | 3,051 (86.33) | |
| Yes | 2,036 (10.39) | 483 (13.67) | |
| **Recurrence within 10 years** | | | 0.3953 |
| No | 16,289 (83.12) | 2,917 (82.54) | |
| Yes | 3,307 (16.88) | 617 (17.46) | |

MET; metabolic equivalent

**Table S6.** Multivariable logistic regression models for reduction in body weight among obese patients.

| | OR | 95% CI | *P* |
|---|---|---|---|
| **Cardiac rehabilitation** | 1.108 | 0.980-1.252 | 0.1015 |
| **Sex** | | | |
| Male | 0.842 | 0.759-0.933 | 0.0011 |
| Female | 1 (Ref) | | |
| **Age (years)** | | | |
| <40 | 1 (Ref) | | |
| ≥40 and <50 | 0.866 | 0.591-1.270 | 0.4620 |
| ≥50 and <60 | 0.672 | 0.465-0.973 | 0.0353 |
| >60 and <70 | 0.720 | 0.498-1.041 | 0.0809 |
| ≥70 | 0.914 | 0.631-1.326 | 0.6367 |
| **Residence** | | | |
| Urban | 1 (Ref) | | |
| Rural | 1.089 | 0.973-1.219 | 0.1392 |
| **Income level** | | | |
| Medicaid | 1.085 | 0.862-1.366 | 0.4878 |
| 1st quantile (low) | 1.031 | 0.927-1.146 | 0.5767 |
| 2nd quantile | 1.054 | 0.948-1.173 | 0.3312 |
| 3rd quantile | 1.003 | 0.911-1.105 | 0.9455 |
| 4th quantile (high) | 1 (Ref) | | |
| **Registered disability** | | | |
| None or other disabilities | 1 (Ref) | | |
| Mild (brain/physical/mental) | 1.182 | 1.034-1.352 | 0.0146 |
| Severe (brain/physical/mental) | 1.175 | 0.900-1.533 | 0.2357 |
| **Charlson Comorbidity Index** | | | |
| 0-2 | | | |
| 3-5 | 1.153 | 1.052-1.264 | 0.0023 |
| ≥6 | 1.393 | 1.230-1.577 | <.0001 |
| **Hypertension** | 1.018 | 0.919-1.128 | 0.7305 |
| **Diabetes** | 1.197 | 1.080-1.327 | 0.0006 |
| **Dyslipidemia** | 0.774 | 0.674-0.889 | 0.0030 |
| **Musculoskeletal disorders** | 1.210 | 1.081-1.354 | 0.0009 |
| **Smoking** | | | |
| Never | 1 (Ref) | | |
| Ex-smoker | 0.961 | 0.869-1.064 | 0.4435 |
| Current smoker | 0.735 | 0.658-0.821 | <.0001 |
| **Alcohol consumption** | | | |
| None | 1 (Ref) | | |
| Low risk | 1.030 | 0.944-1.123 | 0.5081 |
| Moderate risk | 0.980 | 0.751-1.279 | 0.8812 |
| High risk | 1.177 | 0.914-1.517 | 0.2063 |
| **Exercise volume (METs·min/week)** | | | |
| ≥3000 | 0.838 | 0.732-0.960 | 0.0105 |
| ≥600 and <3000 | 0.974 | 0.901-1.053 | 0.5152 |
| <600 | 1 (Ref) | | |
| **Readmission before 28 days after discharge** | 1.262 | 1.131-1.408 | <.0001 |
| **Recurrence within 10 years** | 0.981 | 0.889-1.083 | 0.7006 |

MET; metabolic equivalent

**Table S7.** Comparison between smokers who continued and stopped smoking after coronary revascularization.

| | Continued smoking (N=1,087), N (%) | Stopped smoking (N=1,313), N (%) | *P* |
|---|---|---|---|
| **Cardiac rehabilitation** | | | 0.0374 |
| No | 988 (90.89) | 1,159 (88.27) | |
| Yes | 99 (9.11) | 154 (11.73) | |
| **Sex** | | | 0.7351 |
| Male | 1,059 (97.42) | 1,282 (97.64) | |
| Female | 28 (2.58) | 31 (2.36) | |
| **Age (years)** | | | 0.0079 |
| <40 | 37 (3.40) | 44 (3.35) | |
| ≥40 and <50 | 172 (15.82) | 279 (21.25) | |
| ≥50 and <60 | 406 (37.35) | 492 (37.47) | |
| >60 and <70 | 348 (32.01) | 364 (27.72) | |
| ≥70 | 124 (11.41) | 134 (10.21) | |
| **Residence** | | | 0.0016 |
| Urban | 972 (89.42) | 1,219 (93.05) | |
| Rural | 115 (10.58) | 91 (6.95) | |
| Missing data | | 3 | |
| **Income level** | | | <.0001 |
| Medicaid | 32 (2.97) | 20 (1.54) | |
| 1st quantile (low) | 245 (22.75) | 203 (15.68) | |
| 2nd quantile | 217 (20.15) | 224 (17.30) | |
| 3rd quantile | 309 (28.69) | 348 (26.87) | |
| 4th quantile (high) | 274 (25.44) | 500 (38.61) | |
| Missing data | 10 | 18 | |
| **Registered disability** | | | 0.6747 |
| None or other disabilities | 1,005 (92.46) | 1,226 (93.37) | |
| Mild (brain/physical/mental) | 63 (5.80) | 66 (5.03) | |
| Severe (brain/physical/mental) | 19 (1.75) | 21 (1.60) | |
| **Charlson Comorbidity Index** | | | 0.0079 |
| 0-2 | 407 (37.44) | 573 (43.64) | |
| 3-5 | 513 (47.19) | 566 (43.11) | |
| ≥6 | 167 (15.36) | 174 (13.25) | |
| **Hypertension** | | | 0.5291 |
| No | 244 (22.45) | 309 (23.53) | |
| Yes | 843 (77.55) | 1,004 (76.47) | |
| **Diabetes** | | | 0.0016 |
| No | 896 (82.43) | 1,143 (87.05) | |
| Yes | 191 (17.57) | 170 (12.95) | |
| **Dyslipidemia** | | | 0.0143 |
| No | 72 (6.62) | 123 (9.37) | |
| Yes | 1,015 (93.38) | 1,190 (90.63) | |
| **Musculoskeletal disorders** | | | 0.0277 |
| No | 1,012 (93.10) | 1,250 (95.20) | |
| Yes | 75 (6.90) | 63 (4.80) | |
| **Alcohol consumption** | | | 0.0101 |
| None | 476 (43.79) | 535 (40.75) | |

| | | | |
|---|---|---|---|
| Low risk | 510 (46.92) | 689 (52.48) | |
| Moderate risk | 58 (5.34) | 43 (3.27) | |
| High risk | 43 (3.96) | 46 (3.50) | |
| **Exercise volume (METs·min/week)** | | | 0.0333 |
| ≥3000 | 70 (6.44) | 107 (8.15) | |
| ≥600 and <3000 | 490 (45.08) | 634 (48.29) | |
| <600 | 527 (48.48) | 572 (43.56) | |
| **Body mass index (kg/m²)** | | | 0.3314 |
| <18.5 | 20 (1.84) | 18 (1.37) | |
| 18.5-22.9 | 263 (24.20) | 287 (21.86) | |
| 23-24.9 | 275 (25.30) | 328 (24.98) | |
| ≥25 | 529 (48.67) | 680 (51.79) | |
| **Readmission before 28 days after discharge** | | | 0.1375 |
| No | 987 (90.80) | 1,168 (88.96) | |
| Yes | 100 (9.20) | 145 (11.04) | |
| **Recurrence within 10 years** | | | <.0001 |
| No | 898 (82.61) | 1,166 (88.80) | |
| Yes | 189 (17.39) | 147 (11.20) | |

MET; metabolic equivalent

**Table S8.** Multivariable logistic regression models for cessation of cigarette smoking.

| | OR | 95% CI | *P* |
|---|---|---|---|
| **Cardiac rehabilitation** | 1.181 | 0.896-1.556 | 0.2376 |
| **Sex** | | | |
| Male | 0.904 | 0.515-1.588 | 0.7265 |
| Female | 1 (Ref) | | |
| **Age (years)** | | | |
| <40 | 1 (Ref) | | |
| ≥40 and <50 | 1.297 | 0.795-2.117 | 0.2982 |
| ≥50 and <60 | 1.073 | 0.668-1.723 | 0.7698 |
| >60 and <70 | 1.091 | 0.672-1.774 | 0.7241 |
| ≥70 | 1.044 | 0.610-1.787 | 0.8759 |
| **Residence** | | | |
| Urban | 1 (Ref) | | |
| Rural | 0.652 | 0.484-0.877 | 0.0047 |
| **Income level** | | | |
| Medicaid | 0.437 | 0.235-0.814 | 0.0091 |
| 1st quantile (low) | 0.480 | 0.375-0.613 | <.0001 |
| 2nd quantile | 0.593 | 0.464-0.759 | <.0001 |
| 3rd quantile | 0.618 | 0.497-0.769 | <.0001 |
| 4th quantile (high) | 1 (Ref) | | |
| **Registered disability** | | | |
| None or other disabilities | 1 (Ref) | | |
| Mild (brain/physical/mental) | 0.953 | 0.661-1.373 | 0.7943 |
| Severe (brain/physical/mental) | 1.118 | 0.586-2.130 | 0.7356 |
| **Charlson Comorbidity Index** | | | |
| 0-2 | 1 (Ref) | | |
| 3-5 | 0.934 | 0.774-1.128 | 0.4806 |
| ≥6 | 1.101 | 0.793-1.529 | 0.5650 |
| **Hypertension** | 1.073 | 0.874-1.317 | 0.5019 |
| **Diabetes** | 0.728 | 0.546-0.970 | 0.0300 |

| | | | |
|---|---|---|---|
| **Dysipidemia** | 0.724 | 0.527-0.996 | 0.0486 |
| **Musculoskeletal disorders** | 0.830 | 0.569-1.210 | 0.3321 |
| **Alcohol consumption** | | | |
| None | 1 (Ref) | | |
| Low risk | 1.084 | 0.906-1.296 | 0.3786 |
| Moderate risk | 0.596 | 0.389-0.913 | 0.0175 |
| High risk | 0.936 | 0.595-1.469 | 0.7724 |
| **Exercise volume (METs·min/week)** | | | |
| ≥3000 | 1.270 | 0.907-1.778 | 0.1632 |
| ≥600 and <3000 | 1.084 | 0.911-1.292 | 0.3635 |
| <600 | 1 (Ref) | | |
| **Body mass index (kg/m²)** | | | |
| <18.5 | 0.973 | 0.489-1.935 | 0.9380 |
| 18.5-22.9 | 1 (Ref) | | |
| 23-24.9 | 1.024 | 0.806-1.302 | 0.8459 |
| ≥25 | 1.075 | 0.866-1.333 | 0.5128 |
| **Readmission before 28 days after discharge** | 1.215 | 0.919-1.606 | 0.1717 |
| **Recurrence within 10 years** | 0.679 | 0.530-0.869 | 0.0021 |

MET; metabolic equivalent
